# Spatiotemporal Dynamics of Hand-Foot-Mouth Disease and Its Relationship with Meteorological Factors in Jiangsu Province, China

**DOI:** 10.1371/journal.pone.0131311

**Published:** 2015-06-29

**Authors:** Wendong Liu, Hong Ji, Jun Shan, Jin Bao, Yan Sun, Juan Li, Changjun Bao, Fenyang Tang, Kun Yang, Robert Bergquist, Zhihang Peng, Yefei Zhu

**Affiliations:** 1 Key Lab of Enteric Pathogenic Microbiology, Ministry of Health, Jiangsu Provincial Center for Disease Control and Prevention, Nanjing, China; 2 Jiangsu Meteorological Service Center, Nanjing, China; 3 Jiangsu Meteorological Observatory, Nanjing, China; 4 Department of Schistosomiasis Control, Jiangsu Institute of Parasitic Diseases, Wuxi, China; 5 Independent Researcher, Ingeröd 407, Brastad, Sweden; 6 Department of Epidemiology & Biostatistics, School of Public Health, Nanjing Medical University, Nanjing, China; University of Rochester, UNITED STATES

## Abstract

Hand, foot and mouth disease (HFMD) is an important public health issue in mainland China, including Jiangsu Province. The main purpose of this study was to depict the epidemiological characteristics of HFMD and evaluate the effects of meteorological variables on its dynamics via spatiotemporal analytic methods, which is essential for formulating scientific and effective prevention and control strategies and measures. In total, 497,910 cases of HFMD occurred in the 2009-2013 period, with an average annual incidence of 126.3 per 100,000 in Jiangsu. Out of these, 87.7% were under 5 years old with a male-to-female incidence ratio of 1.4. The dominant pathogens of the laboratory-confirmed cases were EV71 and CoxA16, accounting for 44.8% and 30.6% of all cases, respectively. Two incidence peaks were observed in each year, the higher occurring between April and June, the lower between November and December. The incidence ranged between 16.8 and 233.5 per 100,000 at the county level. The incidence in the South of the province was generally higher than that in the northern regions. The most likely spatiotemporal cluster detected by space–time scan analysis occurred in May-June of 2012 in the southern region. Average temperature and rainfall were positively correlated with HFMD incidence, while the number of days with rainfall ≥ 0.1mm, low temperature, high temperature and hours of sunshine were negatively related. Particularly, relative humidity had no relationship. In conclusion, the prevalence of HFMD in Jiangsu Province has an obvious feature of seasonality. The etiological composition changed dynamically and might be a latent driving force for the temporal variation of the incidence of HFMD. A moderately warm environment promotes the transmission of the HFMD viruses, while particularly cold and hot climate conditions restrain their transmission.

## Introduction

Hand, foot and mouth disease (HFMD) is a common human syndrome caused by highly contagious, intestinal viruses of the Picornaviridae family，which include coxsackieviruses, echoviruses, and enteroviruses (EVs)[1]. It usually affects infants and children younger than 5 years old. In recent decades, the disease has become a substantial burden throughout eastern and southeastern Asia, causing major public health concerns worldwide. In particular, the epidemic situation of HFMD in China is quite serious. The estimated incidence of HFMD is 1.2 per 1,000 person-years in the 2010–2012 period in China, and the disease is responsible for 500–900 reported deaths every year, mainly in young children[2].

The incidence of HFMD varies with respect to spatial and temporal distribution, which has been demonstrated in many affected countries and regions. For example, it peaked in June in northern China, whereas semi-annual outbreaks in May and September–October in the South[2]. HFMD peaks were observed during the summer season in Taiwan[3] and Hongkong[4], and in March or May in Singapore[5]. Seasonal and geographical variation could be related to climate, as suggested by some studies[2,6,7]. For instance, in China, the observed high incidence of HFMD in the East and South has been found to be associated with low latitudes and various meteorological factors, including temperature, vapor pressure, duration of sunshine, relative humidity, population density and movements of people, such as immigration[2]. In Singapore, a significant association between the weekly HFMD incidence and weekly temperatures and rainfall with a 1–2 weeks lag time has been found [5].

The present study was designed to ascertain the epidemiological features, viral aetiology and effect of meteorological variables on the incidence of HFMD through 2009–2013 in Jiangsu Province, Eastern China, with special emphasis on elucidation of the dynamics and immunity patterns of local HFMD and with a view to optimize interventions.

## Materials and Methods

### Study area

Jiangsu is one of the eastern coastal provinces in mainland China, which is located between longitudes 116°21′-121°54′E and latitudes 30°46′-35°08′N neighboring with Shandong in the North, Anhui in the West with Zhejiang and Shanghai in the South. It has 109 counties under the jurisdiction of 13 prefectures, covers a territory of more than 100,000 km^2^ and has a resident population of about 80 million (See S1 Fig).

### Case definition

The diagnosis of HFMD was carried out as guided according to the criteria issued by National Health and Family Planning Commission of the People’s Republic of China. Cases with maculopapular or vesicular rash on hand, foot, mouth, and buttock with or without fever were characterized as mild forms of HFDM, while neurological complications (aseptic meningitis, encephalitis, encephalomyelitis, acute flaccid paralysis, or dysfunction of the autonomic nervous system), cardiopulmonary complications (pulmonary oedema, pulmonary haemorrhage, or cardiorespiratory failure) or both were characterized as severe; Laboratory confirmation of HFMD was completed by RT-PCR, real-time PCR, or virus isolation using throat swabs or stool specimens. It was worth mentioning that we carefully conducted cleansing database, including the deletion of suspected cases, data logic checks and duplication of cases deletion.

### Data sources

Information regarding HFMD in Jiangsu Province during 2009–2013, including sex, age, postal address, date of illness onset, case type(mild or severe) as well as type of pathogen(for laboratory-determined cases) was obtained from China information system for disease control and prevention (http://www.cdpc.chinacdc.cn). Demographic data were collected from the Scientific Data Sharing Center of Public Health (http://www.phsciencedata.cn/Share/index.jsp). Monthly meteorological data for each county for the period 2009–2013 were downloaded from China Meteorological Data Sharing Service System (http://cdc.cma.gov.cn).

### Statistical analysis

Descriptive statistics was used to illustrate the characters of the population distribution and temporal distribution of HFMD. SAS software version 8.1 (SAS Institute Inc., Cary, NC, United States) was employed to investigate the level of statistical significance. The age-standardized incidence was calculated for each county, and smoothed maps of Empirical Bayes Kriging were produced in ArcGIS software version 10.0 (ESRI, Redlands, CA, USA) to state the spatial patterns of HFMD and its aetiology composition[3].

### Space-time scan statistic

A retrospective, space-time scan statistic[8,9] was conducted with SaTScan software version 9.0 (http://www.satscan.org/) to determine whether or not HFMD cases were randomly distributed over space and time in Jiangsu Province. In this approach, the spatial aspect is governed by the circular scan window, which constitutes the bottom of a cylinder, whose height corresponds to the time dimension. In this way, it is possible to move in space and time with variable size and location, not only scanning the whole study area, but also covering the entire study period, in principle using an infinite number of scanning windows. The null hypothesis is that disease risk is the same inside the window as outside it, while the alternative is that the risk is elevated within the window compared to the outside. For each window, a log likelihood ratio (LLR) is calculated and tested and a p-value estimated through Monte Carlo simulation. The window with the maximum LLR is defined as the most likely cluster, that is, the cluster least likely to be due to chance. Other windows with a statistically significant LLR are considered as secondary clusters. In this study, a Poisson distribution of HFMD was assumed for the calculation of the LLRs. The spatial size of scanning window was limited to 20% of the total population at risk and the length of time limited at 6 months. The number of Monte Carlo replications was set at 999.

### Bayesian model

Bayesian statistical approaches[10]were used to examine the spatiotemporal patterns of HFMD, and its relationships with the climatic variables. All meteorological variables for this approach were standardized by subtracting the arithmetic mean calculated from each county and then dividing by the standard deviation (SD).

We assumed that the number of observed cases *O*_*it*_ in county *i* and month *t* follows a Poisson distribution, i.e. *O*_*it*_ ~ Poisson (*μ*_*it*_). We introduced the covariates spatial and spatiotemporal effects on the log transformation of *μ*_*it*_, i.e. *log*(*μ*_*it*_) and developed three models for *log*(*μ*_*it*_):

No spatiotemporal effects: $log(\mu_{it})=log(E_{it})+\alpha +\sum_{k}\beta_{k}X_{itk}$ Model 1

Spatiotemporal independent effects: $log(\mu_{it})=log(E_{it})+\alpha +\sum_{k}\beta_{k}X_{itk}+u_{j}+v_{t}$ Model 2

Spatiotemporal interactive effects: $log(\mu_{it})=log(E_{it})+\alpha +\sum_{k}\beta_{k}X_{itk}+u_{jt}$ Model 3

In each model, *E*_*it*_ represents the expected HFMD case number in county *i* at month *t*, *α* the intercept, *β*_*k*_ the regression coefficient and *X*_*itk*_ the covariate. In model 2, *u*_*j*_ and *v*_*t*_ represent county-specific and month-specific effects, respectively, while *u*_*j*_ is a random term that allows for spatially structured variation and *v*_*t*_ a random term representing variation between months.

The spatial correlation of *u* was assumed to have a multivariate normal distribution, *u* ~MVN (0, ∑) with the variance-covariance matrix ∑. The spatial process was assumed to be an isotropic stationary process with ∑ defined as an exponential correlation function, ∑_*ij*_ = *σ* ^2^ exp(−*ρd*_*xy*_), where *d*_*xy*_ is the Euclidean distance between county *x* and *y* (shortest straight-line), *σ* the spatial variance, while *ρ* is a smoothing parameter that controls the rate of correlation decay with increasing distance and measures the range of geographical dependency.

The Bayesian models were fitted in WinBUGS (MRC Biostatistics Unit, Cambridge, UK). The deviance information criterion (DIC) was used to compare the goodness-of-fit of the models, the smallest DIC indicating the best fit[11,12].

### Ethics statement

This study was approved by the Ethics Committee at the Jiangsu Provincial Center for Disease Control and Prevention, China. As the data about HFMD cases were downloaded from existing online databases, it was complete impracticality to obtain the informed consent.

## Results

### Demographic characteristics

Totally 497,910 cases of HFMD occurred in the period 2009–2013 in Jiangsu Province reaching an average annual incidence of 126.3 per 100,000. Out of these cases, 87.7% were younger than 5 years. Among the different age groups of population, the one year olds had the highest incidence rate of all, i.e. 3,250.8 per 100,000. The number of cases, in particular the severe cases sharply increased from 6 month, while gradually declining after the age of 3 had been reached. There was a significant difference in incidences between genders (χ2 = 24933.05, *p*<0.0001),with a male-to-female incidence ratio of 1.4. A total of 5,976 cases were diagnosed as severe HFMD during the 5 study years, accounting for 1.2% of all cases. The fatality rate was 0.9% of those with severe illness (52 cases) (Table 1).

**Table 1:** Population distribution of HFMD during 2009–2013 in Jiangsu province, China.

Age	Number of HFMD			Incidence of HFMD			Number of severe HFMD			Death of severe HFMD		
	male	female	total	male	female	total	male	female	total	male	female	total
**0 year**	21505	12683	34188	1057.43	697.53	887.54	409	186	595	11	2	13
**0–5 months**	2094	1153	3247				44	12	56	0	0	0
**6–11 months**	19411	11530	30941				365	174	539	11	2	13
**1 year**	83431	50884	134315	3760.02	2660.06	3250.77	1418	810	2228	19	7	26
**2 years**	68533	41742	110275	3184.89	2246.49	2750.06	877	511	1388	3	5	8
**3 years**	58989	36944	95933	2808.48	2036.35	2450.63	626	319	945	4	1	5
**4 years**	37715	24064	61779	1818.79	1341.11	1597.2	275	197	472	0	0	0
**5–9 years**	33395	22599	55994	310.15	234.15	274.23	192	132	324	0	0	0
**10–14 years**	2262	1572	3834	27.97	23.58	25.99	16	6	22	0	0	0
**> = 15 years**	760	832	1592	0.45	0.49	0.47	2	0	2	0	0	0
**Total**	306590	191320	497910	154.37	97.86	126.34	3815	2161	5976	37	15	52

Altogether, 21,375 cases were laboratory-confirmed between 2009 and 2013, accounting for 4.3% of total cases. The predominated pathogens were EV71 and Cox A16, accounting for 44.8% and 30.6% of the laboratory-confirmed cases, respectively. There was a significant difference between the pathogens in the mild cases and the severe cases (χ2 = 1723.93, *p*<0.0001) with the proportion of EV71 79% in the severe cases against 31% in the mild cases. Notably, the proportion of EV71 was as high as 94% in those who died by the infection. There were also differences between age groups (χ2 = 244.82, *p*<0.0001), the proportions of EV71 in those under 10 were a little higher than in the other groups (Table 2).

**Table 2:** Etiological constitution of laboratory confirmed HFMD during 2009–2013 in Jiangsu province, China.

Variables	Etiological constitution			Number of laboratory-confirmed cases
	Cox A16	EV71	other EV	
Total	30.57	44.84	24.59	21375
** Mild case**	34.49	39.19	26.31	18340
** Severe case**	6.85	78.95	14.2	3035
** Died cases**	0	94.12	5.88	34
**Age group**				
** 0 year**	26	45.52	28.49	1327
** 0–5 months**	22.12	48.67	29.2	113
** 6–11 months**	26.36	45.22	28.42	1214
** 1 year**	26.52	44.51	28.96	5776
** 2 years**	30.54	45.38	24.08	4804
** 3 years**	31.87	45.12	23.02	4258
** 4 years**	36.3	42.69	21.01	2694
** 5–9 years**	33.98	46.64	19.38	2322
** 10–14 years**	32.17	39.86	27.97	143
**> = 15 years**	39.22	35.29	25.49	51

### Characteristics in temporal distribution

**Fig 1:**
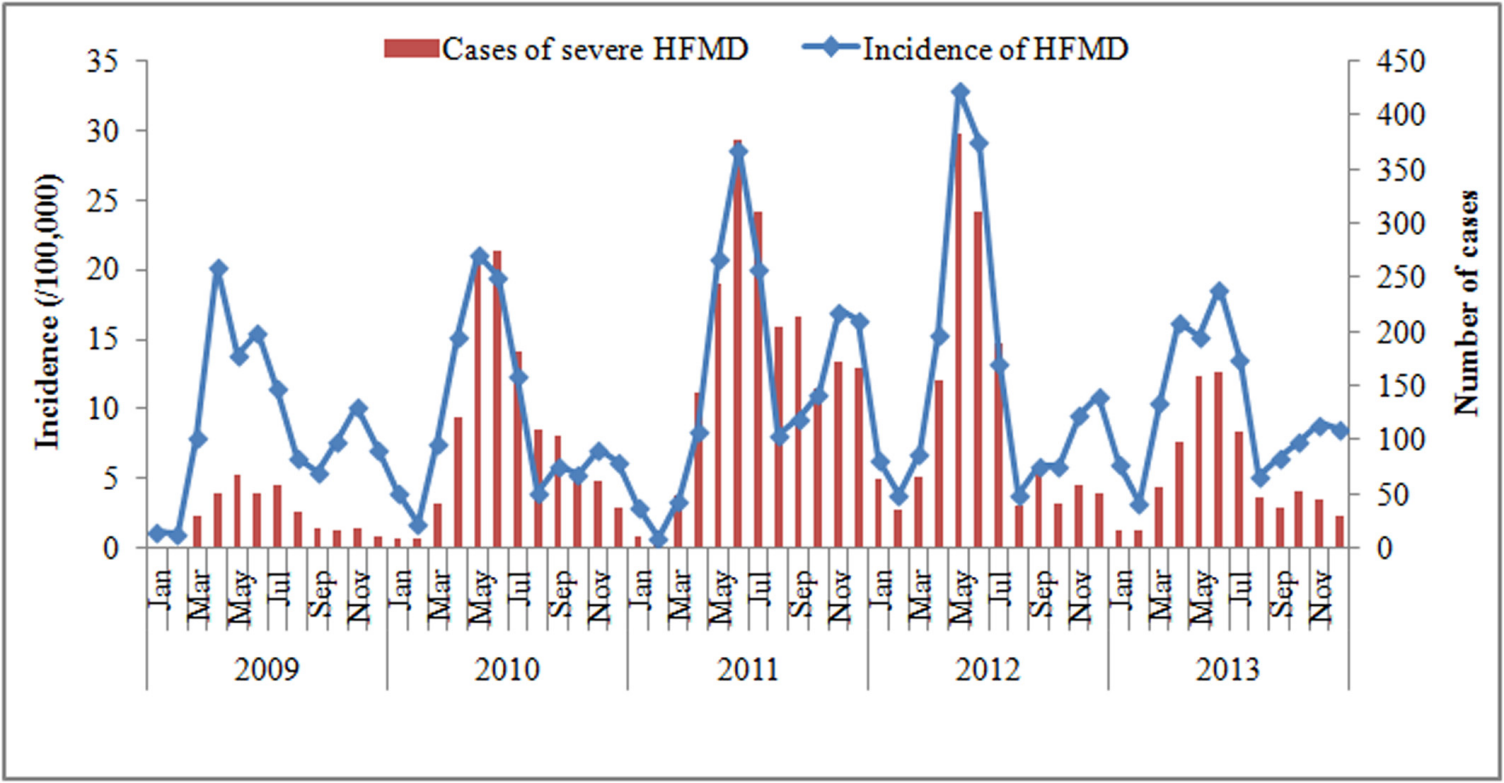
Monthly distribution of incidence of HFMD and cases of severe HFMD during 2009–2013 in Jiangsu province, Eastern China.

The incidence of HFMD gradually increased year by year from 2009 through 2012, but decreased in 2013 by 16.7%. The number of severe HFMD declined even more sharply in 2013 (43.6%). Two incidence peaks were observed in each year, the higher between April and June and the lower between November and December. It is worth mentioning that just one peak of severe HFMD was observed and its period was coincided with the main peak of the disease (Fig 1).

The etiological composition of Laboratory confirmed cases changed dynamically over time. The ratios of CoxA16 and EV71 infections remained around 80% during 2009–2012, while the ratio of other EV viruses slightly increased year by year and sharply increased from 18.3% in 2012 to 52.1% in 2013, accompanied by a substantial decline in the proportion of CoxA16 (from 41.0 to 12.0%).

Notably, the monthly proportion of EV71 varied seasonally in a characteristic way with two peaks, one in April-July and the other in October-December, respectively, which was consistent with the incidence peaks of HFMD. The variation of the monthly ratio of the other EVs had no obvious seasonality but when its monthly ratio reached the highest level, the incidence of HFMD was usually at a relative low level. No significant pattern was discovered in the variation of monthly ratio of the CoxA16 viruses.

### Characteristics of spatial distribution

**Fig 2:**
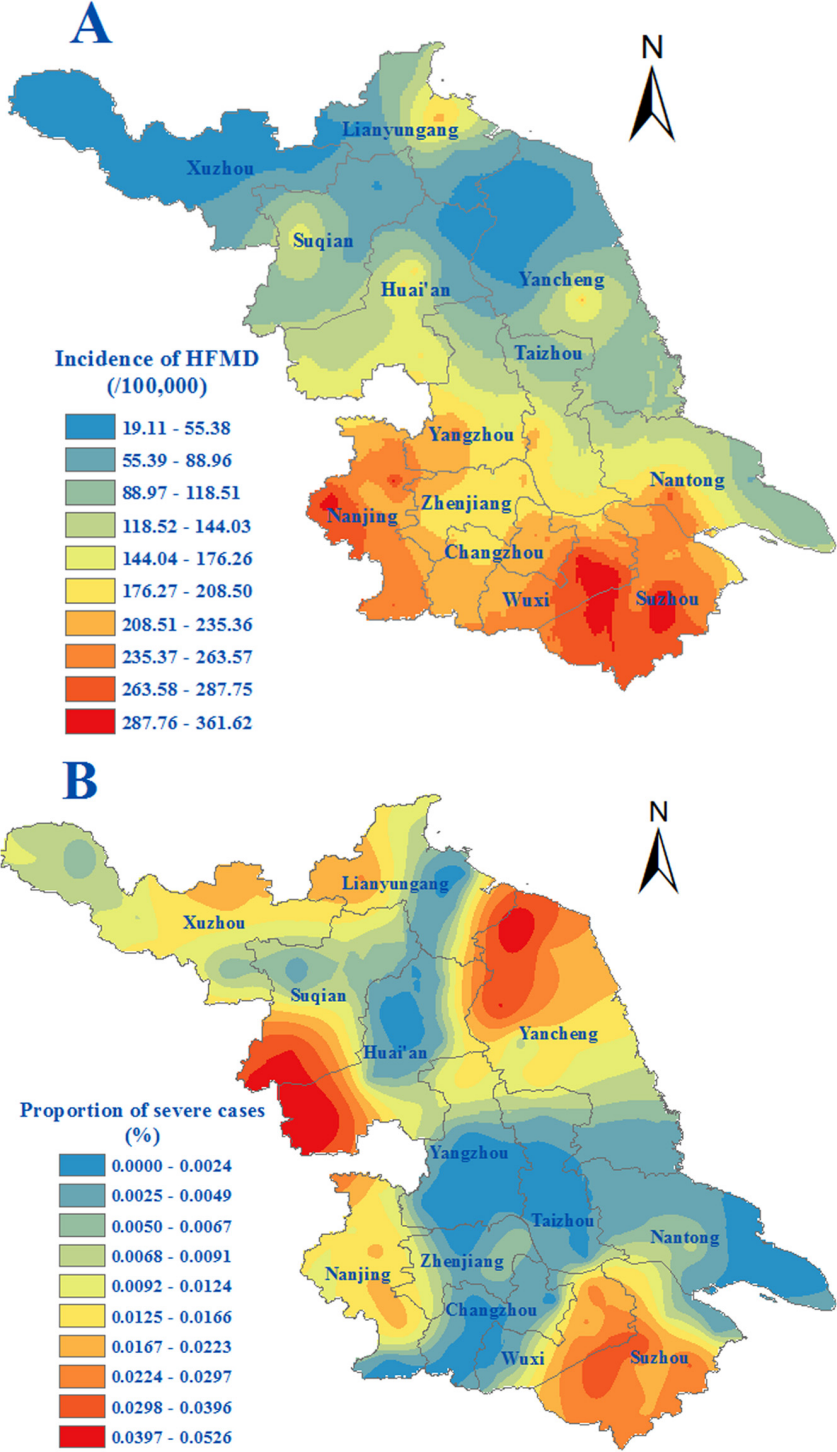
Spatial distribution of HFMD during 2009–2013 in Jiangsu province, Eastern China. A. Spatial distribution of age-standardized incidence of HFMD. B. Spatial distribution of proportion of severe HFMD cases.

**Fig 3:**
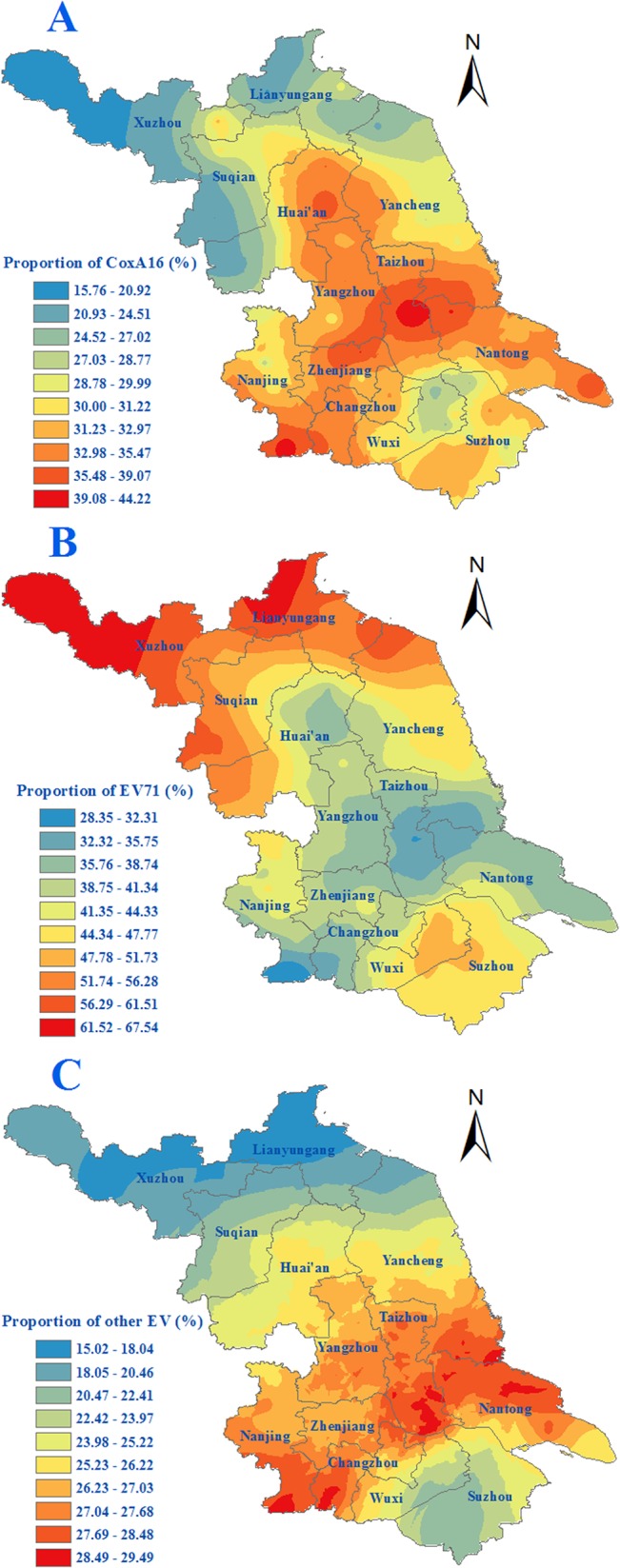
Spatial distribution of proportion of EV71, CA16 and the other EVs HFMD during 2009–2013 in Jiangsu province, Eastern China. A. Spatial distribution of proportion of CA16 of HFMD cases. B. Spatial distribution of proportion of EV71 of HFMD cases. C. Spatial distribution of proportion of the other EVs of HFMD cases.

The age-standardized incidence ranged between 19.11 and 361.62 per 100,000 at the county level. Counties located in southern regions, namely Suzhou, Wuxi, Changzhou and Nanjing, usually had an age-standardized incidence of more than 150 per 100,000. Some counties in the northern regions, i.e. Yancheng, Huaian, Suqian and Lianyungang, had relatively high incidence. The severe cases were mainly in Suzhou, Huaian and Suqian. Wuxi and Nanjing were also found to be prone to harbor relatively high numbers of severe cases. Whereas the geographical proportion of severe cases to the total HFMD cases were the highest in Huaian and Suqian followed by Yancheng, the ratio in the southern regions was lower. (Fig 2). The etiological composition of the infections varied with EV71 dominating in the northern regions and other EVs in the central part of the province. In the southern regions, however, there was no dominant virus serotype (Fig 3).

### Spatiotemporal cluster analysis

**Fig 4:**
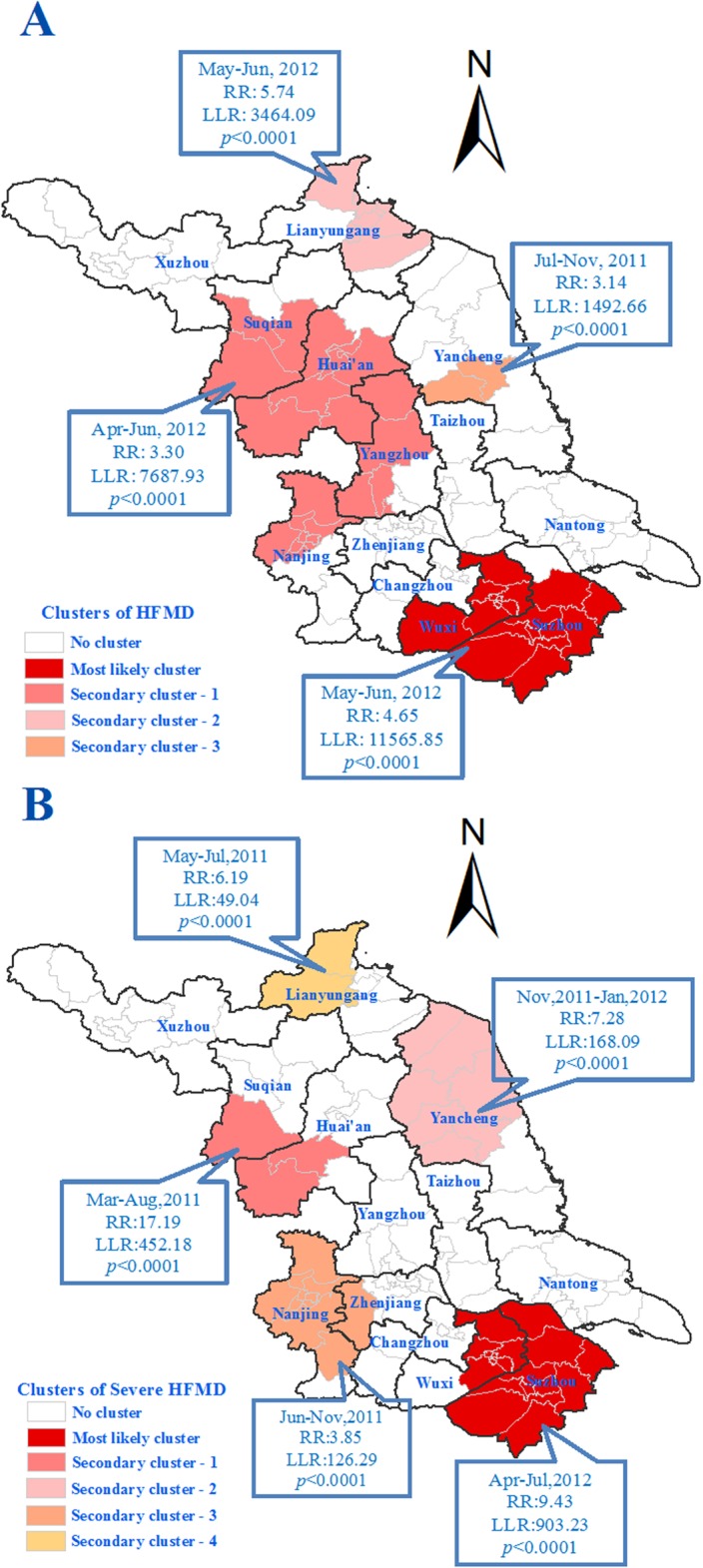
This is the Fig 4 Spatiotemporal clusters of HFMD during 2009–2013 in Jiangsu province, China. A. Clusters of total HFMD cases. B. Clusters of severe cases.

Four spatiotemporal clusters of total cases were detected by space-time scan statistic analysis. All the clusters occurred in the period of the first incidence peak in 2012, except the 3^rd^ secondary cluster, which occurred between July and November in 2011. The most likely cluster had 15,576 cases with a relative risk (RR) of 4.65. It covered 18 counties in Wuxi and Suzhou, two southern prefectures of Jiangsu Province. The 1^st^ secondary cluster had 15,700 cases with a RR of 3.30 and covered 24 counties, including 9 in Nanjing, 5 in Yangzhou, 7 in Huai’an and 3 in Suqian. The 2^nd^ secondary cluster had 3,772 cases with a RR of 5.74 and covered 7 counties in Lianyungang, a northern prefecture of Jiangsu province. The 3^rd^ secondary cluster had 3,232 cases with a RR of 3.14. It covered just 2 counties in Yancheng. Moreover, five clusters of severe cases were detected (Fig 4).

### Effect of meteorological factors on the epidemics

Three Bayesian models, i.e. one without spatiotemporal effects (model 1), one with independent spatial and temporal effects (model 2) and another with interactive spatiotemporal effects (model 3), were fitted with the same 7 meteorological variables. Model 3 had the smallest DIC value and was therefore selected as the final model for further analysis, which suggested that the prevalence of HFMD was spatially autocorrelated, the degree of which changed over time. According to the parameter estimation of model 3, relative humidity had no relationship with the incidence of HFMD as the 95% confidence interval of its coefficient including zero. Average temperature and rainfall were positively correlated with the incidence of HFMD, while the other variables, namely number of days with rainfall ≥ 0.1mm, lowest temperature, highest temperature and hours of sunshine, were negatively related to HFMD incidence (Table 3).

**Table 3:** Parameters estimation and their 95% confidence intervals (in brackets) of Bayesian models for county level HFMD prevalence data during 2009–2013 in Jiangsu province, China.

Parameter	Model 1	Model 2	Model 3
**Intercept**	-0.089(-0.091, -0.086)	-0.184(-0.493, 0.082)	-0.314(-0.338, -0.292)
**Rainfall**	-0.070(-0.075, -0.065)	0.047(0.040, 0.054)	0.082(0.033,0.134)
**Number of days with rainfall ≥ 0.1mm**	0.104(0.099,0.110)	-0.041(-0.050, -0.031)	-0.145(-0.185, -0.101)
**Average temperature**	9.09(8.935, 9.255)	3.255(3.010, 3.503)	5.673(4.263,7.038)
**Lowest temperature**	-6.296(-6.383, -6.208)	-1.798(-1.970, -1.629)	-3.432(-4.227, -2.646)
**Highest temperature**	-2.380(-2.461, -2.302)	-0.657(-0.794, -0.521)	-2.149(-2.814, -1.475)
**Hours of sunshine**	-0.278(-0.283, -0.274)	-0.067(-0.079, -0.055)	-0.122(-0.173, -0.070)
**Relative humidity**	0.017(0.014, 0.021)	0.014(0.011, 0.018)	0.023(-0.006, 0.048)
**Spatial correlation**		2.233(1.676, 2.867)	
**Temporal correlation**		2.135(1.438, 2.960)	
**DIC**	487300	208800	45200

## Discussion

HFMD is a common infectious disease, which has been endemic in dozens of countries since last 70’s. In recent years, the outbreaks of HFMD in the Asian-Pacific region, especially the epidemic induced by EV71 in Taiwan in 1998, attracted global attention [13,14]. HFMD is also an important public health issue in mainland China[2], and it was made a notifiable infectious disease of Class C, after the pandemic in the spring of 2008[15]. Thus, comprehensive understanding of the epidemiological characteristics of this disease is essential for formulating scientific and effective prevention and control strategies.

Nearly half million of HFMD cases occurred in Jiangsu province in the last 5 years, with an average annual incidence of 126.3 per 100,000 in the whole population, which was lower than that of the surrounding provinces[16], but somewhat higher than the national incidence as well as that in many countries or regions where HFMD is endemic. The incidence in males was found to be higher than in females, and so was the ratio of severe cases and mortality, and this suggests that some factors increase male susceptibility at the host genetic level, host immune status and/or behavior patterns. However, it may also be due to reporting bias. The prevalence was found to be in sharp disequilibrium among the different age groups. Nearly 90% cases were concentrated in those under 5 years of age, in agreement with some other reports[17,18]. According to the demographic characteristics, the groups under 5 years old, especially those aged between 6 months and three years, were the focus for the prevention and control, precisely the target population for the EV71 vaccine which has just been developed [19]and expected to be put into production shortly. The acceptability of EV71 vaccine may be compromised as it was only to prevent the severe disease, but might not be able to reduce the incidence of HFMD in children caused by other pathogens. Therefore, the best strategy to against HFMD is to develop combined CA16/EV71 vaccines or multivalent vaccines, which could protect children from the attack of EV71, CA16 and other EVs.

Some researchers have pointed out that the prevalence of HFMD presents a periodic nature[4,20]. In our study, a sharp decline was observed in 2013, which followed the four years’ rising trend. We can’t yet conclude whether it suggests a periodic fluctuation due to insufficient observation, and more surveillance efforts should be made to confirm this epidemiological characteristic. Nonetheless, it is clear that the incidence of HFMD in Jiangsu province has seasonality. Two incidence peaks of the general cases occurred in each year, the main in spring and the second in autumn, while the severe cases just showed one peak in spring.

Based on epidemiology and aetiology surveillance from 2009 to 2013 in Jiangsu, it showed that EV71 predominated in laboratory-confirmed severe cases, particularly in fatal cases. Furthermore, the etiological composition of HFMD was also changing dynamically, which might be the latent driving force to the temporal variation of the incidence of HFMD. Firstly, the changing of the monthly proportion of EV71 presented a seasonal pattern consistent with that of the incidence of HFMD. Secondly, once the other EV dominant, the incidence of HFMD was usually in a low prevalence level as showed in the annually fluctuation as well as the seasonal variation of the HFMD epidemic, which may be owing to the basic reproduction number for EV71 was higher than that of CoxA16, and other EVs[21]. Based on these associations, the dynamics of the etiological composition of HFMD could serve as a predictor of the epidemics and transmission dynamics of HFMD outbreaks, thus it is necessary to strengthen the pathogenic surveillance, which could formulate public health measures for controlling the disease.

According to our study, the spatial distribution of incidence of HFMD was also uneven. The incidence in southern regions was generally higher than that in northern regions, which is worthy of conducting a special research to understand the underlying causes to employ more targeted control strategies for this disease. Notably, Suzhou, Wuxi and Nanjing were the main epidemic areas, which suggested that the efforts of surveillance and prevention should be strengthened in these regions. However, some northern counties in Suqian, Huaian and Yancheng should also be paid close attention.

The relationship between meteorological factors and the prevalence of HFMD has been of particular interest for researchers, and some reports have proved that environmental temperature and relative humidity leads to the seasonal variation of HFMD[22–24]. In this study, we evaluated the effects of some meteorological variables on the incidence of HFMD via a spatiotemporal Bayesian model. According to the results, the monthly average temperature was strongly positive association with the incidence of HFMD with a correlation coefficient of 5.673, while the lowest and highest temperature were both negatively related to its prevalence. This suggested that moderately warm environment promotes the transmission of the viruses of HFMD, while the cold and hot climate conditions restrain their transmission, which may be due to two reasons. Firstly, environmental temperature is related to behavioral patterns, and warm weather leads to increased contact among young children, accordingly facilitating the spread of HFMD infection. On the other hand, moderate temperature may also be good for the survival of HFMD viruses in the environment, thereby enhancing their transmission, which needs to be confirmed by further systematic, experimental studies. Thus, the environmental temperature might be one of the major underlying factors causing the spatiotemporal patterns of this disease. Another discovery is that the rainfall favors the prevalence, but too many days of rainfall present the opposite effect. And it is not conducive to the epidemic if the sunshine time is too long. It is worth mentioning that the effects of the latter three variables were far less than that of environmental temperature. Particularly, it was found that relative humidity had nothing to do with the prevalence of this disease, which was different from some other researches[6,22].This would be mainly due to the difference in methodology. Chang HL et al[6] employed Poisson regression analysis and case-crossover methodology to evaluate the association between weather variability and the incidence of EV71 infection. In their research, the study area was considered as a whole, the spatial correlation and heterogeneity were ignored. In another research [22], geographically weighted regression (GWR) models were used to explore the associations between the selected factors and HFMD incidence based on just one month’s data. The seasonal changing of HFMD incidence and climate variables were not taken into consider. In our study, both spatial and temporal variability of the independent and explanatory variables were taken into the Bayesian model, which could more comprehensively reflect the association between the HFMD epidemic and the climate conditions. Notably, these associations might be wrong due to ecological fallacy, and they needs to be further validated by epidemiological experiments.

## Conclusions

In brief, we described the detailed spatiotemporal dynamics of HFMD and of its relationships with meteorological factors from 2009 to 2013 in Jiangsu province, China. The prevalence of HFMD in Jiangsu had an obviously seasonally feature. The southern regions were the focus of prevention and control. The etiological composition changing dynamically and might be the latent driving force to the temporal variation of the epidemic. Some meteorological parameters, especially the environmental temperature maybe another driving factors for the spatiotemporal pattern of HFMD. The moderately warm environment promotes the transmission of the viruses of HFMD, while the cold or hot climate conditions restrain their transmission. The results will be used to predict the forthcoming prevalence and severity of HFMD and serve as reference and basis for the surveillance and control of this disease in the future.

## Supporting Information

S1 Fig Jiangsu province and its location in mainland China. (TIF)
