# Supplementary figures and images for: Spatiotemporal Dynamics of Hand-Foot-Mouth Disease and Its Relationship with Meteorological Factors in Jiangsu Province, China
Source: PLoS One. 2015 Jun 29;10(6):e0131311. doi: 10.1371/journal.pone.0131311 (PMC4488144; doi:10.1371/journal.pone.0131311)

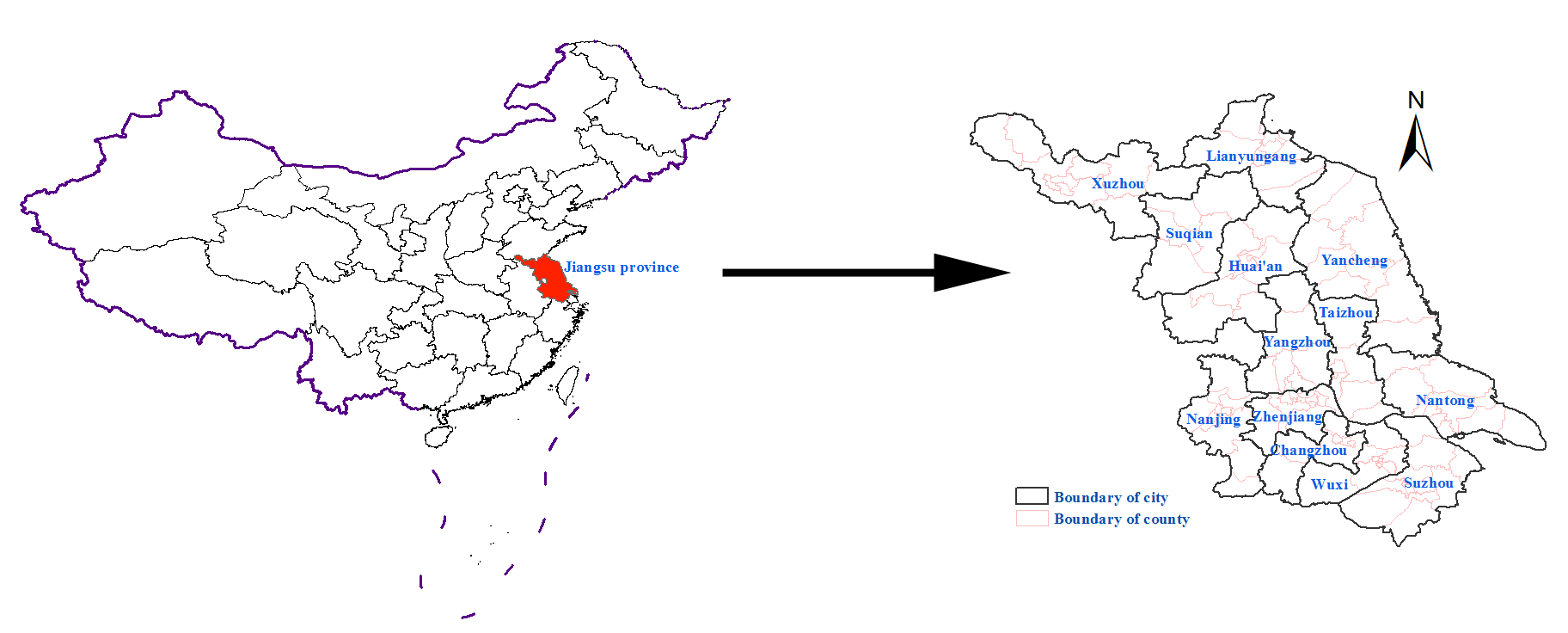

Supplement: S1 Fig — (TIF) [file pone.0131311.s001.tif]
